# Multitarget and Multipathway Regulation of Zhenqi Fuzheng Granule against Non-Small Cell Lung Cancer Based On Network Pharmacology and Molecular Docking

**DOI:** 10.1155/2022/5967078

**Published:** 2022-11-17

**Authors:** Yanqing Zhou, Chenxi Wu, Xian Qian, Jin Zhou, Chengjian Li, Yang Jiao, Yue Yue Li, Liang Zhao

**Affiliations:** ^1^Department of Pharmacy, Shanghai Baoshan Luodian Hospital, Shanghai 201908, China; ^2^College of Pharmacy, Guangxi Medical University, Nanning 530021, China; ^3^Department of Pharmacy, Shanghai Ninth People's Hospital, Shanghai JiaoTong University School of Medicine, Shanghai 200011, China

## Abstract

*Background and Objective*. The morbidity and mortality rates of non-small cell lung cancer (NSCLC) remain high. Zhenqi Fuzheng (ZQFZ) granule, which consists of *Astragali Radix* and *Ligustri Lucidi Fructus*, is commonly used to improve the immunity of cancer patients. However, the mechanism of ZQFZ granule against NSCLC is still unclear. In this study, the network pharmacology and molecular docking approaches were used to investigate the potential mechanism of ZQFZ granule on NSCLC. *Methods*. The ingredients in the ZQFZ granule were considered in one study based on UPLC, and the potential targets were predicted in the SwissTargetPrediction database. NSCLC targets were gathered from GeneCards, OMIM, and TTD databases. The ingredient-target-NSCLC network was drawn by Cytoscape. The protein–protein interaction was obtained from the STRING database, and the gene function and biological pathways were analyzed by Metascape. AutoDock Vina was used to verify the molecular docking between the key compounds and core targets, and PyMol visualized the results. *Results*. 244 targets were related to 13 candidate compounds and 1904 targets were related to NSCLC, of which a total of 106 anti-NSCLC targets were predicted. The compound-target-NSCLC network indicated that sinapinic acid, ferulic acid, asiatic acid, pratensein, and glycitein might be the key components for treating NSCLC. The 41 vital targets (out of 106 targets) above the median calculated by PPI degree were selected for bioinformatics analysis. The top 10 targets out of 41 ranked by MCC were IL-6, SRC, CTNNB1, STAT3, CASP3, TNF, EGFR, MAPK8, HSP90AA1, and PTGS2. ZQFZ granule treatment for NSCLC involved many pathways through KEGG analyses, which included pathways in cancer (hsa05200), proteoglycans in cancer (hsa05205), endocrine resistance (hsa01522), microRNAs in cancer (hsa05206), PI3K-Akt signaling pathway (hsa04151), and IL-17 signaling pathway (hsa04657). Molecular docking studies revealed that sinapinic acid, ferulic acid, asiatic acid, pratensein, and glycitein had good infinity with most core targets. *Conclusions*. This study indicated that ZQFZ granule with multicompounds could treat NSCLC through multitargets and multipathways.

## 1. Introduction

GLOBOCAN 2020 showed that there were an estimated 19 million new cancer cases and 10 million deaths worldwide in 2020. The cancer morbidity and mortality rates remained high, with breast cancer ranking first among women and lung cancer being the leading cause of cancer death [1]. Surgery, radiotherapy, chemotherapy, targeted therapy, immunotherapy, and adjuvant therapy with Chinese medicine are the common means of cancer treatment. It is well known that Chinese medicine compounding has a long history with multicomponent and multitarget characteristics. In complementary treatment, Chinese medicine compounding has a great effect on increasing chemotherapy drugs' chemosensitivity, reducing adverse drug reactions and toxicity, relieving patients' pain, and improving life quality particularly [2].

As a traditional Chinese medicine (TCM), ZQFZ granule has the ability to improve immunity and protect bone marrow and adrenal cortex functions [3]. Combined with surgery, chemotherapy, and radiotherapy, ZQFZ granule could effectively reduce adverse effects caused by chemotherapy and promote recovery of immune function, subsequently improving the patient's quality of life and ultimately decreasing the recurrence rate. A prospective, open-label, randomized controlled trial confirmed that TCM treatment could promote NSCLC patients' quality of life, relieve symptoms, and reduce adverse events. The TCM was composed of the immuno-boosting drug called Kangai injection, herbal decoction, and ZQFZ capsule [4].

ZQFZ granule consists of *Astragali Radix* (AsR, dried root of *Astragalus membranaceus*, known as HuangQi in Chinese) and *Ligustri Lucidi Fructus* (LLF, dried ripe fruit of *Ligustrum lucidum*, known as NvZhenZi in Chinese). Triterpenoids and phenylethanoid glycosides are the two major types of constituents present in AsR [5]. As one of the most commonly used herbal remedies for cancer in China, AsR can activate immune regulation, inhibit cell proliferation, and attenuate adverse effects caused by cytotoxic therapy [6]. Similarly, the chemical constituents of LLF mainly include saponins, flavonoids, and nitrogen-containing chemicals [7]. Studies have shown that LLF can enhance the sensitivity of adriamycin-induced apoptosis, improve immunity, and promote apoptosis, so as to inhibit tumor growth [8]. The classical formulation of ZQFZ is a (2 : 1, w/w) mixture of AsR and LLF. The mixture was decocted twice with 10–30 times water, and the filtrate was concentrated into a thick paste, mixed evenly with sucrose, and dried into granules [9].

**Figure 1:**
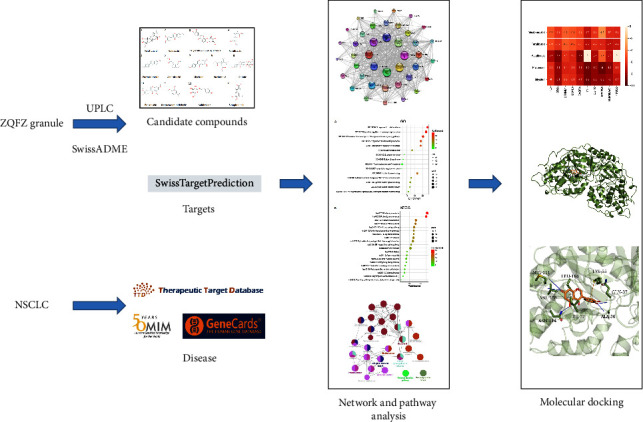
The flowchart.

However, due to the complexity of the components of Chinese herbal compounds, the mechanisms and targets of reaction are difficult to elucidate. The mechanisms are being studied by examining the impacts of monomeric components, which are not exhaustive. Based on the whole perspective, network pharmacology combines network biology and multipharmacology to compensate for the shortage of multicomponent system research of TCM. In this study, we focused primarily on the active components from a recent study based on ultra-performance liquid chromatography (UPLC) [10] and explored the molecular mechanism of ZQFZ granule for the treatment of NSCLC. The flowchart is shown in Figure 1.

## 2. Materials and Methods

### 2.1. Collection of the Main Ingredients from ZQFZ Granule and ADME Evaluation

The ingredients in ZQFZ granule were considered from one study based on UPLC [10]. The main active compounds were selected according to Lipinski's rule of five (RO5) [11]. The criteria of Lipinski's RO5 were as follows: a molecule weight (MW) should be less than 500, the number of hydrogen bond donors (Hdon) should be less than 5, the number of hydrogen bond acceptors (Hacc) should be less than 10, lipid-water partition coefficient (LogP) no more than 5, and the number of rotatable bonds (Rbon) no more than 10. The chemical structures and canonical SMILES were searched in PubChem (https://pubchem.ncbi.nlm.nih.gov/) and the ADME of compounds were uploaded into SwissADME (https://www.swissadme.ch/) to evaluate the druggability.

### 2.2. Screening of Targets of the Vital Ingredients from ZQFZ Granule in NSCLC

The SwissTargetPrediction (https://www.swisstargetprediction.ch/) was used to predict the potential targets of vital ingredients from the ZQFZ granule. The targets with a probability greater than zero were included. NSCLC targets were gathered from GeneCards (https://www.genecards.org/), OMIM (https://omim.org/), and TTD (https://db.idrblab.net/ttd/) by the keywords “non-small cell lung cancer” and “non-small cell lung carcinoma.” The common genes, probably the targets for ZQFZ granule to treat NSCLC, were assessed by Venny 2.1.0 (https://bioinfogp.cnb.csic.es/tools/venny/). An ingredient-disease-target network was visualized by Cytoscape 3.7.2 software.

### 2.3. Protein–Protein Interaction (PPI) Analysis and Vital Target Screening

The PPI of common genes was analyzed in STRING (https://string-db.org/). The organism was set to *Homo sapiens* and the confidence of 0.4 was selected as significant. The active interaction sources for PPI analysis and median calculation included known, predicted, and other interactions. The PPI network was analyzed and visualized using the Cytoscape software. The node size represents the degree value and the edge represents the connection between proteins. In this study, the vital genes, which were above the median calculated by degree, were then used for biological function analysis. Moreover, the top 10 proteins ranked by maximal clique centrality (MCC) using the cytoHubba plug-in were selected as core targets.

### 2.4. Gene Ontology (GO) and Kyoto Encyclopedia of Genes and Genomes (KEGG) Pathways Enrichment Analyses

GO analysis of the vital genes, including the biological process (BP), cellular component (CC), and molecular function (MF), was enriched and analyzed by the Metascape database (https://metascape.org/gp). The screening thresholds were a minimum of 3 genes, *p* < 0.01 and an enrichment factor greater than 1.5. The same method and settings were used for KEGG pathway enrichment. Finally, the results were plotted as bubble charts using the online bioinformatics tool (https://bioinformatics.com.cn/). Furthermore, the Cytoscape plug-in ClueGo was used for analyzing the relevant KEGG pathway.

### 2.5. Molecular Docking

To verify the binding of the vital ingredients of ZQFZ granule with predicted core genes, compounds in mol2 format were downloaded from TCMSP (https://tcmsp-e.com/) and the core protein structures were acquired from RCSB PDB (https://www.rcsb.org/). The compounds and proteins were imported to AutoDockTools 1.5.6 software for dehydration, hydrogenation, and other pretreatments. Then, molecular docking for analyzing the binding activity was performed using AutoDock Vina 1.1.2 [12] software, and some great results were visualized using PyMol 4.6.0 software.

## 3. Results

### 3.1. The Chemical Structure and ADME Properties of the Vital Ingredients from ZQFZ Granule

**Figure 2:**
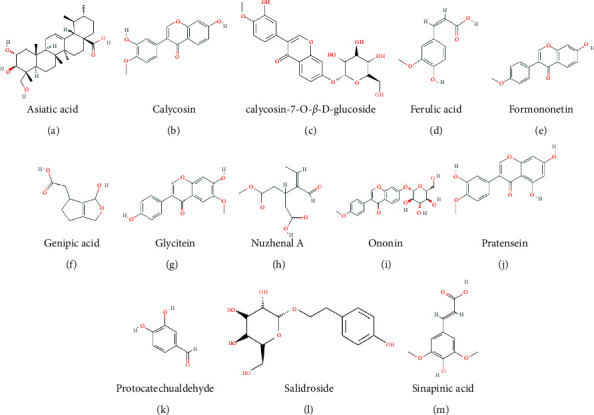
Structures of the 13 candidate compounds extracted from ZQFZ granule.

A recent study has identified 95 chemical components from ZQFZ granule including 15 batches from 3 producers based on UPLC [10]. The chemical structures of the compounds were searched in the PubChem database, and ADME properties were evaluated in SwissADME. A total of 15 compounds met Lipinski's RO5, indicating that these components had good drug-like properties. Moreover, potential target genes were predicted based on the chemical structure via the SwissTargetPrediction database, and two of them had no targets. Therefore, we selected 13 compounds (Figure 2 and Table 1) as candidate compounds.

### 3.2. Acquisition of Targets of the Candidate Ingredients from ZQFZ Granule in NSCLC

**Figure 3:**
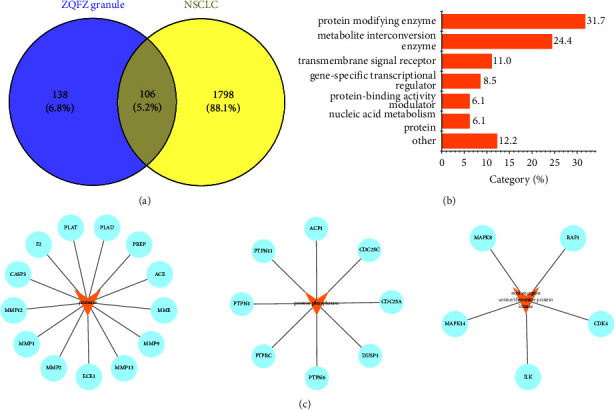
Targets of the candidate ingredients from ZQFZ granule in NSCLC. (a) A Venn diagram applied to acquire the 106 common targets between the 13 candidate compounds from the ZQFZ granule and NSCLC. (b) Panther classification of the 106 common targets. (c) Targeted proteins involved in protein-modifying enzymes (PC00260).

Based on the SwissTargetPrediction database, a total of 244 targets for the 13 candidate compounds were acquired. In addition, results from GeneCards (https://www.genecards.org/), Online Mendelian Inheritance in Man (OMIM, https://omim.org/), and Therapeutic Target Database (TTD, https://db.idrblab.net/ttd/) for non-small cell lung cancer identified 1904 targets relevant to NSCLC. Furthermore, the Venn diagram reflected the common 106 target genes of the candidate compounds and NSCLC for further analysis (Figure 3(a)). The 106 common targets of ZQFZ granule against NSCLC are listed in Supplementary Table S1. Moreover, these 106 target proteins were grouped into seven different classes according to cellular function via PANTHER (https://www.pantherdb.org/), with protein-modifying enzyme (PC00260, 31.7%) being the most enriched class (Figure 3(b)). These protein-modifying enzymes include protease (PC00190), which covers ACE, CASP3, ECE1, F2, MME, MMP1, MMP12, MMP13, MMP2, MMP9, PLAT, PLAU, and PREP, protein phosphatase (PC00195) that covers ACP1, CDC25A, CDC25C, DUSP1, PTPN1, PTPN11, PTPN6, and PTPRC, and nonreceptor serine/threonine protein kinase (PC00167) including CDK4, ILK, MAPK14, MAPK8, and RAF1 (Figure 3(c)). The results above implied that the candidate ingredients from the ZQFZ granule exerted anti-NSCLC effects through a variety of targets and biological functions.

### 3.3. Construction of a Candidate Ingredients from the ZQFZ Granule Target NSCLC Network

**Figure 4:**
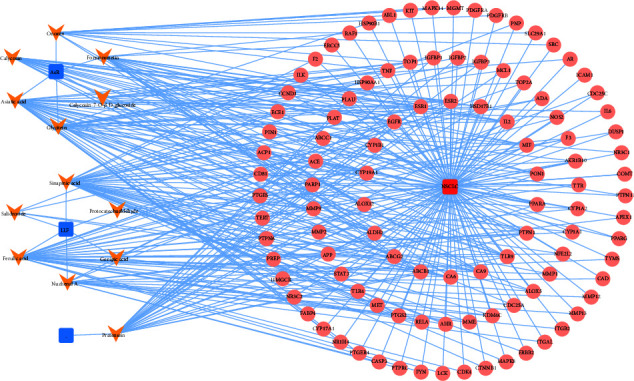
Potential compounds from the ZQFZ granule target NSCLC network. Blue nodes represent the herbs derived from the ZQFZ granule, orange nodes show the vital ingredients from herbs, and pink nodes show shared targets between potential targets of the vital compounds from the ZQFZ granule and NSCLC targets. ZQFZ granule, Zhenqi Fuzheng granule; AsR, *Astragali Radix*; LLF, *Ligustri Lucidi Fructus*; —, not detected in LLF or AsR but detected in ZQFZ; NSCLC, non-small cell lung cancer.

To study the mechanism of ZQFZ granule in the treatment of NSCLC, 106 common targets and 13 candidate compounds were used for constructing the potential ZQFZ granule target NSCLC network. As shown in Figure 4, all compounds were associated with multiple targets, generating a total of 344 component-target connections between the 13 compounds and 106 targets. The network revealed sinapinic acid (degree = 40) had the most targets, followed by ferulic acid (degree = 33), asiatic acid (degree = 30), pratensein (degree = 24), and glycitein (degree = 21), indicating that these compounds from ZQFZ granule were highly likely to become key components for treating NSCLC.

### 3.4. Protein–Protein Interaction (PPI) Analysis

**Figure 5:**
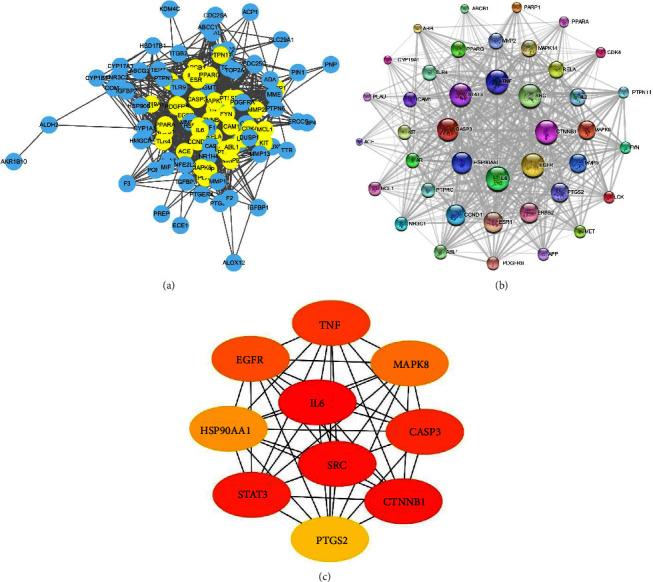
Protein–protein interaction (PPI) network: (a) PPI network of the 106 common targets between the 13 candidate compounds from ZQFZ granule and NSCLC. The PPI was analyzed in the STRING database and visualized by Cytoscape software. Nodes represent target proteins and edges represent interactions among targets. Yellow nodes represent the 41 vital targets above the median calculated by degree. (b) PPI network of the 41 vital targets. The larger the node is, the greater the degree is. (c) The top 10 targets in the PPI network ranked by maximal clique centrality (MCC) using the cytoHubba plug-in.

The 106 common targets of the predicted ZQFZ granule and NSCLC were explored in the STRING database and protein interaction relationships were gained (Figure 5(a)). To further study the relationship between these targets, 41 vital genes above the median calculated by degree were selected and the protein interaction relationships were analyzed via Cytoscape software.  Table 2 shows the details of the 41 vital targets. The PPI network generated 41 nodes, which represent proteins, and 552 edges which represent the connections between proteins. The larger node means the greater degree (Figure 5(b)). Furthermore, the top 10 targets ranked by MCC using the cytoHubba plug-in were IL-6, SRC, CTNNB1, STAT3, CASP3, TNF, EGFR, MAPK8, HSP90AA1, and PTGS2 (Figure 5(c)) and selected as core targets for interaction with NSCLC.

### 3.5. Gene Ontology (GO) and Kyoto Encyclopedia of Genes and Genomes (KEGG) Pathways Enrichment Analyses

**Figure 6:**
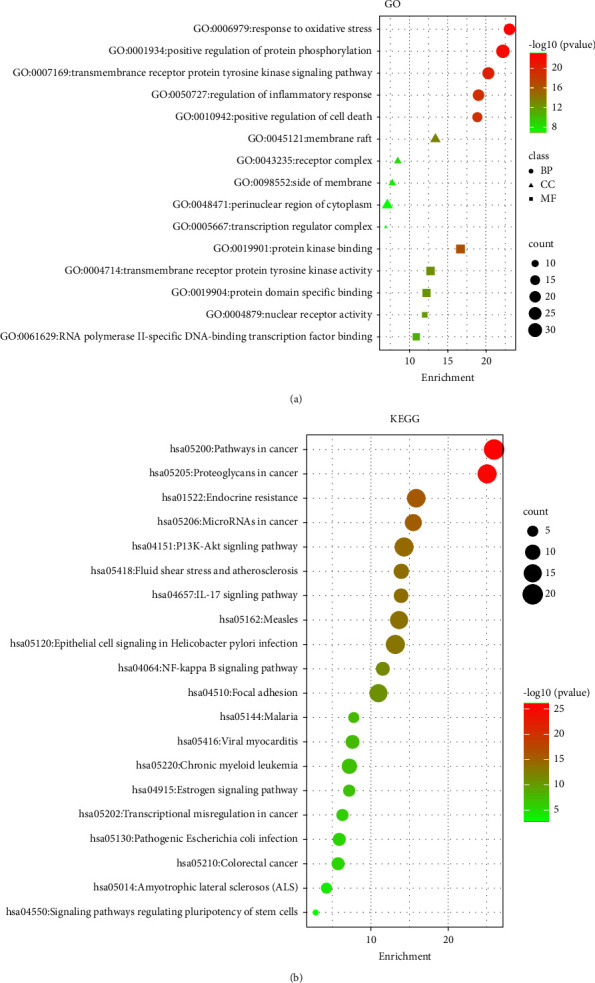
Gene ontology (GO) and Kyoto Encyclopedia of Genes and Genomes (KEGG) pathways enrichment analyses. (a) Bubble chart of the top 5 biological processes (BP), cellular component (CC), and molecular function (MF) terms identified by GO enrichment analysis. (b) Bubble chart of the top 20 pathway terms identified by KEGG pathway enrichment analysis. The size of the dots represents gene numbers. The larger is the dot, the higher is the number of genes.

The 41 vital targets between ZQFZ granule and NSCLC were further analyzed by GO enrichment analysis and KEGG pathway analysis by Metascape. The GO results showed 421 entries for BP, 49 entries for CC, and 82 entries for MF. The top 5 BPs, CCs, and MFs are shown in bubble charts (Figure 6(a)). The top 5 biological processes were related to oxidative stress (GO: 0006979), protein phosphorylation (GO: 0001934), and transmembrane receptor protein-tyrosine kinase signaling pathway (GO: 0007169). Cellular components analysis revealed including membrane raft (GO: 0045121), receptor complex (GO: 0043235), and side of the membrane (GO: 0098552). For molecular functions, the targets were enriched in protein kinase binding (GO: 0019901), transmembrane receptor protein-tyrosine kinase activity (GO: 0004714), and protein domain-specific binding (GO: 0019904). The complex list of the top 5 BP, CC, and MF pathway terms enriched in ZQFZ granule against NSCLC is shown in Supplementary Table S2.

**Figure 7:**
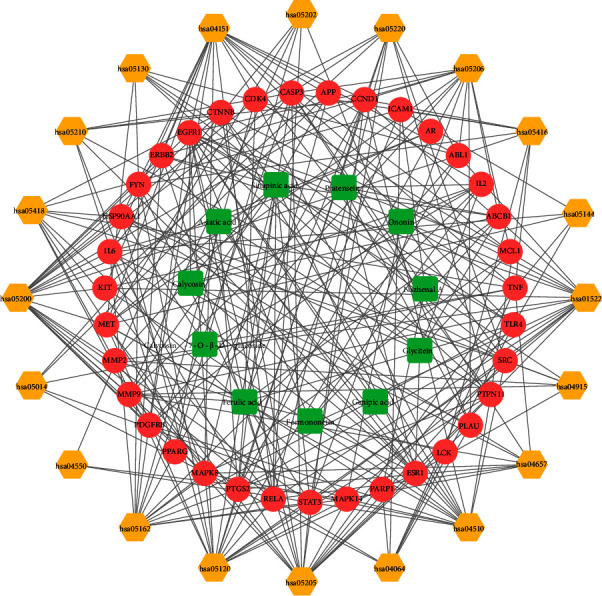
A compound-target-pathway network. The green round rectangle represents the chemical compounds, the pink circles represent the targets, and the yellow hexagon represents the signal pathways.

**Figure 8:**
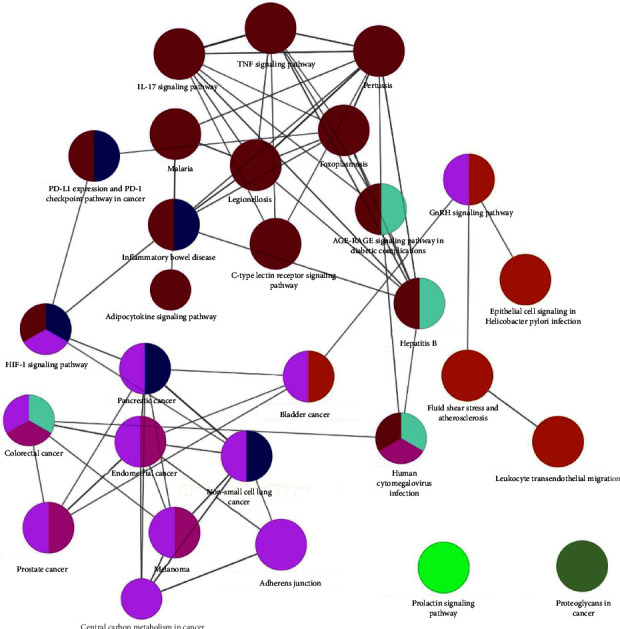
KEGG analysis of the 41 vital targets of ZQFZ granule against NSCLC using the ClueGO plug-in. Each node is a representative enrichment pathway. The nodes imply gene numbers shared between the pathways; the color indicates the enrichment classification of the node.

In addition, the KEGG pathway revealed that 206 pathways were involved, and the bubble chart (Figure 6(b)) reflects the top 20 terms.  Table 3 displays detailed information about the top 20 pathways related to pathways in cancer (hsa05200), proteoglycans in cancer (hsa05205), endocrine resistance (hsa01522), microRNAs in cancer (hsa05206), PI3K-Akt signaling pathway (hsa04151), and IL-17 signaling pathway (hsa04657). In particular, 8 out of 10 core targets (IL-6, CTNNB1, STAT3, CASP3, EGFR, MAPK8, HSP90AA1, and PTGS2) involved in the first-term pathways in cancer (hsa05200), and 7 core targets (IL-6, SRC, CTNNB1, STAT3, CASP3, TNF, and EGFR) involved in the second-term proteoglycans in cancer (hsa05205). A compound-target-pathway network (Figure 7) shows the connection between the top 20 pathways, targets, and compounds. The predictive diagram of the mechanism of the proteoglycans pathway is displayed in Figure 8.

### 3.6. Molecular Docking Simulation

**Figure 9:**
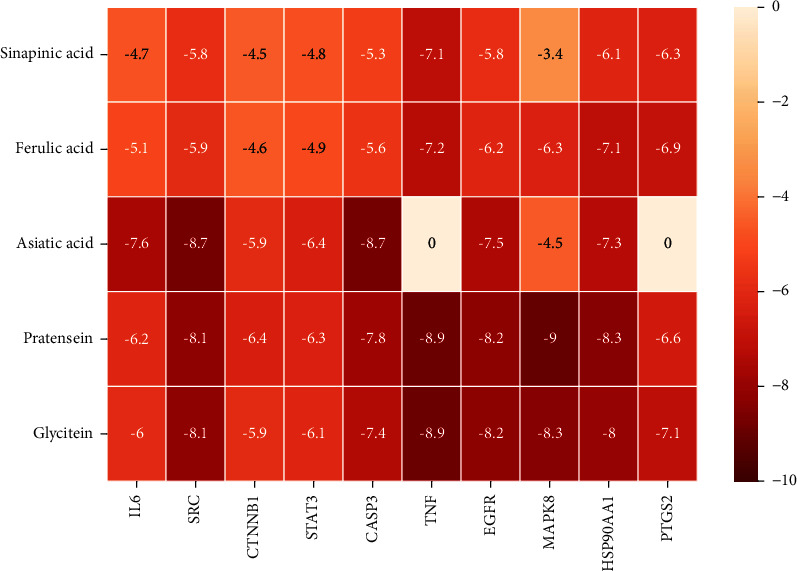
Heat map of the affinity values via molecular docking. Molecular docking of core targets with the top 5 compounds ranked by degree. The darker the box is, the stronger the bond is.

According to the potential compounds from the ZQFZ granule target NSCLC network (Figure 4), sinapinic acid, ferulic acid, asiatic acid, pratensein, and glycitein had a high number of targets against NSCLC and were the top 5 compounds ranked by degree. The PDBIDs and resolutions of the core target proteins are listed in Supplementary Table S3. Thus, molecular docking predicted the binding of the five compounds to core targets related to NSCLC. The affinity is usually regarded as the binding effect of the compound to the target; the lower the absolute value of affinity is, the more stable the binding is. As shown in Figure 9, sinapinic acid showed a strong binding to TNF, with an affinity value of −7.1 kcal/mol; ferulic acid showed a strong binding to TNF and HSP90AA1, with affinity values of −7.2 and −7.1 kcal/mol; asiatic acid showed the strong binding to IL-6, SRC, CASP3, EGFR, and HSP90AA1 with affinity values of −7.6, −8.7, −8.7, −7.5, and −7.3 kcal/mol; pratensein showed the strong binding to SRC, CASP3, TNF, EGFR, MAPK8, and HSP90AA1 with affinity values of −8.1, −7.8, −8.9, −8.2, −9.0, and −8.3 kcal/mol; and glycitein showed a strong binding to SRC, CASP3, TNF, EGFR, MAPK8, HSP90AA1, and PTGS2, with affinity values of −8.1, −7.4, −8.9, −8.2, −8.3, −8.3, and −7.1 kcal/mol. In general, an affinity value of less than −4.25 kcal/mol means some binding capacity, the affinity value of less than −5.0 kcal/mol means good binding capacity, and the affinity value of less than −7.0 kcal/mol means strong binding capacity.  Table 4 shows some representative docking results with the core targets.

As shown in Table 4, the structure of asiatic acid could form one hydrogen bond, two hydrogen bonds, and two hydrogen bonds with Tyr103, Tyr32, and Asp56, respectively, in IL-6. Respectively, asiatic acid could interact with Leu273 and Asp404 via one hydrogen bond in SRC. The structure of pratensein could interact with Asp207 and Lys281 through one hydrogen bond in CTNNB1. The structure of asiatic acid could form one hydrogen bond and two hydrogen bonds with Glu638, and Gln644, respectively, in STAT3. The structure of asiatic acid could form one hydrogen bond with Arg207, Phe250, and Phe256, respectively, in CASP3. Sinapinic acid could form one hydrogen bond with Tyr195 and Tyr227 in TNF. Glycitein could interact with Lys745 and Gly796 through one hydrogen bond in EGFR. The structure of pratensein could interact with Ala36, Gln37, and Asn114 through one hydrogen bond, respectively, and Met111 via two hydrogen bonds in MAPK8. The structure of ferulic acid was linked to Asn51, Tyr139, and Trp162 through one hydrogen bond, respectively, in HSP90AA1. One hydrogen bond could combine pratensein with Tyr139 in HSP90AA1. Glycitein could interact with His90, Tyr385, and Arg513 via one hydrogen bond, respectively, and Ser530 via two hydrogen bonds in PTGS2. Furthermore, some known inhibitors for the core targets were used to validate the molecular docking. It is shown in Table 5. The result indicated the affinities of the inhibitors were comparable to the respective compounds of ZQFZ granule. The structures of the known inhibitors for the core targets are listed in Supplementary Table S4.

## 4. Discussion

Lung cancer remains the leading cause of cancer death, accounting for 18% of all cancer deaths [1]. To explore the active ingredients and potential mechanisms of ZQFZ granule for the treatment of NSCLC, this study comprehensively investigated the therapeutic effect of ZQFZ granule in NSCLC.

Generally, compounds for network pharmacology studies are obtained from public databases, while the effect of the content may be ignored, resulting in inaccurate predictions. Especially, active compounds were derived from the study that analyzed 15 batches from 3 producers, which significantly increased the credibility of data [10]. Based on Lipinski's RO5 and SwissTargetPrediction databases, 13 compounds were selected as candidate compounds and 106 potential targets were screened against NSCLC. Moreover, a potential compound from the ZQFZ granule target NSCLC network implied that sinapinic acid, ferulic acid, asiatic acid, pratensein, and glycitein were probably the critical components for NSCLC treatment. Sinapinic acid, one of the most common hydroxycinnamic acids, has an anticancer effect on prostate cancer cells. Treatment with sinapinic acid (1 mM for 72 h) could eliminate half the number of PC-3 and LNCaP cells [13]. Ferulic acid, as a 4-hydroxy-3-methoxycinnamic acid, can inhibit phorbol-12-myristate-13-acetate (PMA)-stimulated invasion of A549 cells at a concentration of ≥100 *μ*M [14]. Asiatic acid, as a natural triterpene, has been proven to enhance the sensitivity of multidrug-resistant A549 cells to cisplatin by downregulating P-glycoprotein (MDR1) and its targets [15]. Glycitein had significant cytotoxic effects on gastric cancer cells by inducing cell apoptosis and G0/G1 phase cell cycle arrest via the ROS-related MAPK/STAT3/NF-*κ*B signaling pathway [16]. Therefore, these compounds play an important role in the treatment of tumors. Notably, there are few studies on the treatment of NSCLC with sinapinic acid, pratensein, and glycitein.

The 106 targets in common between ZQFZ granule and NSCLC were considered potential targets. The top 10 core targets shown in Figure 5(c) included IL-6, SRC, CTNNB1, STAT3, CASP3, TNF, EGFR, MAPK8, HSP90AA1, and PTGS2. These target genes are associated with the regulation of tumorigenesis. IL-6, STAT3, and TNF are involved in immunoactivation and play critical roles in the development of NSCLC. CTNNB1 and EGFR are highly expressed in carcinomas such as lung and breast cancers. SRC is related to endocrine regulation and CASP3 is commonly regarded as the predominant terminal shear enzyme in cell apoptosis. Furthermore, the relationship between the potential compounds and the core targets could be validated by the literature. For instance, sinapinic acid significantly repressed TNF levels and activated CASP3 in acute doxorubicin-induced cardiotoxicity rats [17]. Ferulic acid inhibited UVB-induced TNF and IL-6 protein expression in mice skin tissue, which might be related to the activation of MAPK and NF-kappa B signaling pathways [18]. Asiatic acid induced HT-29 cell apoptosis via CASP3 activation and inhibited the growth and metastasis of breast cancer in mice by downregulating SRC protein expression [19, 20].

The core targets of ZQFZ granule for the treatment of NSCLC were applied to obtain an enrichment map of GO and KEGG pathway analyses via the Metascape database. The results showed BP was involved in oxidative stress, protein phosphorylation, protein-tyrosine kinase signaling pathway, and inflammatory response. CC was primarily concerned with various cell bodies such as membrane, complexes, and cytoplasm. Additionally, MF was mainly associated with protein kinase binding. Furthermore, the main signaling pathways were endocrine resistance, PI3K/Akt, IL-17, and NF-kappa B signaling pathways, while the diseases involved were cancers, atherosclerosis, and measles.

IL-6 is a pleiotropic cytokine with various biological functions in immunity, tissue regeneration, and metabolism. It is highly expressed in lung cancer and negatively correlated with survival [21]. Blocking IL-6 expression can inhibit lung cancer promotion, cell proliferation, angiogenesis markers, and tumor cell-intrinsic STAT3 activation [22]. STAT3 is mainly expressed in naive CD4+ T cells and activated *p*-STAT3 can modulate the transcriptional activity of target genes associated with tumor cell migration and invasion [23]. IL-6/STAT3 signaling is activated in lung tumorigenesis and metastasis. IL-6 can activate STAT3 signaling and escape host immunity by upregulating and recruiting granulocyte-likemyeloid-derived suppressor cells and type II macrophage polarization [24]. EGFR, a member of the receptor tyrosine kinase ERBB family, is an important oncogene in NSCLC progression. EGFR-TKIs are used for treating NSCLC by modulating the immune microenvironment. However, EGFR-TKI resistance is widely present in NSCLC and promotes immune escape via increased PD-L1 expression and, subsequently, activates the PI3K/AKT/mTOR pathway aberrantly [25, 26]. The PI3K/Akt signaling pathway, a classical mediator that regulates cell growth and inflammation, was found to be activated in this study. There are 17 targets identified in this pathway, and IL-6, TNF, EGFR, MAPK8, and HSP90AA1 are core targets. Tumor necrosis factor (TNF), a cytokine, can combine with extracellular death receptors to activate the apoptosis process and regulate the inflammatory microenvironment via the PI3K/Akt pathway in NSCLC [27, 28]. Upregulation of HSP90AA1 is related to poor overall survival in cancer patients [29]. This could be due to the fact that HSP90AA1 overexpression reduces immune surveillance and resists foreign substances [30].

This is a novel way to study the mechanism of ZQFZ granule in the treatment of NSCLC via a network pharmacologic method. The selected compounds of ZQFZ granule were derived from a recent study based on UPLC. The results may be useful to better understand the multipathway and multitarget regulation of ZQFZ granule against NSCLC.

In total, 13 compounds in the ZQFZ granule and 106 common targets against NSCLC were screened by network pharmacology analysis. GO and KEGG enrichment analyses implied the 13 compounds in the ZQFZ granule could treat NSCLC via multiple NSCLC pathological processes. However, the theoretical observations still need to be validated by clinical studies in the future. This study provided a theoretical basis for the clinical application of ZQFZ granule in NSCLC.

## 5. Conclusion

This study revealed the pharmacological mechanism of ZQFZ granule in NSCLC based on network pharmacology and molecular docking. GO and KEGG enrichment analyses implied that the 13 compounds in the ZQFZ granule played an important role in the treatment of NSCLC. This study provided a theoretical basis for further research on the clinical application of ZQFZ granule in NSCLC.

## Figures and Tables

**Table 1:** Pharmacological and molecular properties of the main compounds in ZQFZ granule.

Name	Formula	MW (g/mol)	Rbon	Hacc	Hdon	TPSA (Å)	LogP	LogS	Log Kp (cm/s)	Type	Source
Asiatic acid	C_30_H_48_O_5_	488.7	2	5	4	97.99	3.2	−6.33	−5.23	Triterpenoid	AsR
Calycosin	C_16_H_12_O_5_	284.26	2	5	2	79.9	2.4	−3.57	−6.3	Flavonoid	AsR
Calycosin-7-O-*β*-D-glucoside	C_22_H_22_O_10_	446.4	5	10	5	159.05	2.65	−3.05	−8.57	Flavonoid	AsR
Ferulic acid	C_10_H_10_O_4_	194.18	3	4	2	66.76	1.62	−2.11	−6.41	Organic acid	LLF
Formononetin	C_16_H_12_O_4_	268.26	2	4	1	59.67	2.49	−3.73	−5.95	Flavonoid	AsR
Genipic acid	C_9_H_12_O_4_	184.19	2	4	2	66.76	1.37	−0.21	−8.15	Organic acid	LLF
Glycitein	C_16_H_12_O_5_	284.26	2	5	2	79.9	2.36	−3.57	−6.3	Flavonoid	AsR
Nuzhenal A	C_10_H_14_O_5_	214.21	7	5	1	80.67	1.37	−0.66	−7.66	Others	LLF
Ononin	C_22_H_22_O_9_	430.4	5	9	4	138.82	2.65	−3.18	−8.22	Flavonoid	AsR
Pratensein	C_16_H_12_O_6_	300.26	2	6	3	100.13	2.38	−4.06	−5.93	Flavonoid	—
Protocatechualdehyde	C_7_H_6_O_3_	138.12	1	3	2	57.53	0.79	−1.76	−6.37	Others	LLF
Salidroside	C_14_H_20_O_7_	300.3	5	7	5	119.61	1.04	−0.92	−8.88	Phenylethanoid	LLF
Sinapinic acid	C_11_H_12_O_5_	224.21	4	5	2	75.99	1.63	−2.16	−6.63	Organic acid	LLF

LLF, *Ligustri Lucidi Fructus*; AsR, *Astragali Radix*; —, not detected in LLF or AsR but detected in ZQFZ granule.

**Table 2:** Information on 41 vital targets of ZQFZ granule against NSCLC.

Number	Uniprot ID	Gene symbol	Description	Degree
1	P05231	IL-6	Interleukin-6	40
2	P00533	EGFR	Epidermal growth factor receptor	39
3	P35222	CTNNB1	Catenin beta-1	39
4	P12931	SRC	Proto-oncogenetyrosine-protein kinase Src	39
5	P01375	TNF	Tumor necrosis factor	39
6	P40763	STAT3	Signal transducer and activator of transcription 3	38
7	P42574	CASP3	Caspase-3	37
8	P07900	HSP90AA1	Heat shock protein HSP 90-alpha	35
9	P24385	CCND1	G1/S-specific cyclin-D1	34
10	P03372	ESR1	Estrogen receptor alpha	34
11	P04626	ERBB2	Receptor protein-tyrosine kinase ERBB-2	33
12	P35354	PTGS2	Prostaglandin G/H synthase 2	32
13	P45983	MAPK8	Mitogen-activated protein kinase 8	31
14	P14780	MMP9	Matrix metalloproteinase 9	31
15	P60568	IL2	Interleukin-2	29
16	Q04206	RELA	Nuclear factor NF-kappa B p65 subunit	28
17	Q16539	MAPK14	Mitogen-activated protein kinase 14	28
18	P08253	MMP2	Matrix metalloproteinase 2	27
19	P37231	PPARG	Peroxisome proliferator-activated receptor gamma	27
20	O00206	TLR4	Toll-like receptor 4	26
21	P10721	KIT	Mast/stem cell growth factor receptor kit	25
22	P10275	AR	Androgen receptor	25
23	P08575	PTPRC	Receptor-typetyrosine-protein phosphatase C	25
24	P05362	ICAM1	Intercellular adhesion molecule 1	25
25	P04150	NR3C1	Glucocorticoid receptor	24
26	P00519	ABL1	Tyrosine-protein kinase ABL1	24
27	Q07820	MCL1	Induced myeloid leukemia cell differentiation protein Mcl-1	24
28	P09619	PDGFRB	Platelet-derived growth factor receptor beta	23
29	P05067	APP	Amyloid-beta precursor protein	22
30	P08581	MET	Hepatocyte growth factor receptor	22
31	P06239	LCK	Tyrosine-protein kinase LCK	21
32	P06241	FYN	Tyrosine-protein kinase FYN	21
33	Q06124	PTPN11	Protein-tyrosine phosphatase 2C	20
34	P11802	CDK4	Cyclin-dependent kinase 4	19
35	P09874	PARP1	Poly [ADP-ribose]polymerase-1	19
36	Q07869	PPARA	Peroxisome proliferator-activated receptor alpha	19
37	P08183	ABCB1	ATP-dependent translocase ABCB1	17
38	P35869	AHR	Aryl hydrocarbon receptor	17
39	P11511	CYP19A1	Cytochrome P450 19A1	16
40	P00749	PLAU	Urokinase-type plasminogen activator	16
41	P12821	ACE	Angiotensin-converting enzyme	14

**Table 3:** Top 20 KEGG pathway terms enriched in ZQFZ granule against NSCLC.

Term	Pathway	*P* value	Count	Symbols
hsa05200	Pathways in cancer	1.105*E* − 26	20	ABL1/AR/CCND1/CASP3/CDK4/CTNNB1/EGFR/ERBB2/HSP90AA1/IL-6/KIT/MET/MMP2/MMP9/PDGFRB/PPARG/MAPK8/PTGS2/RELA/STAT3
hsa05205	Proteoglycans in cancer	8.793*E* − 26	17	CCND1/CASP3/MAPK14/CTNNB1/EGFR/ERBB2/ESR1/IL-6/MET/MMP2/MMP9/PLAU/PTPN11/SRC/STAT3/TLR4/TNF
hsa01522	Endocrine resistance	1.345*E* − 16	16	CCND1/CDK4/MAPK14/EGFR/ERBB2/ESR1/MMP2/MMP9/MAPK8/SRC/RELA/STAT3/CTNNB1/KIT/ABL1/PTGS2
hsa05206	MicroRNAs in cancer	3.195*E* − 16	13	ABL1/CCND1/CASP3/EGFR/ERBB2/MCL1/MET/MMP9/PDGFRB/ABCB1/PLAU/PTGS2/STAT3
hsa04151	PI3K-Akt signaling pathway	5.023*E* − 15	17	CCND1/CDK4/EGFR/ERBB2/HSP90AA1/IL2/IL-6/KIT/MCL1/MET/PDGFRB/RELA/TLR4/ABL1/MAPK8/PTPN11/TNF
hsa05418	Fluid shear stress and atherosclerosis	1.152*E* − 14	10	MAPK14/CTNNB1/HSP90AA1/ICAM1/MMP2/MMP9/MAPK8/RELA/SRC/TNF
hsa04657	IL-17 signaling pathway	1.23*E* − 14	9	CASP3/MAPK14/HSP90AA1/IL-6/MMP9/MAPK8/PTGS2/RELA/TNF
hsa05162	Measles	2.238*E* − 14	15	CCND1/CASP3/CDK4/FYN/IL2/IL-6/MAPK8/RELA/STAT3/TLR4/EGFR/ERBB2/MAPK14/MCL1/PTPN11
hsa05120	Epithelial cell signaling in *Helicobacter pylori* infection	6.772*E* − 14	17	CASP3/MAPK14/EGFR/MET/MAPK8/PTPN11/RELA/SRC/CCND1/ESR1/STAT3/ABL1/MMP2/ICAM1/PTGS2/HSP90AA1/FYN
hsa04064	NF-kappa B signaling pathway	2.867*E* − 12	8	PARP1/ICAM1/LCK/PLAU/PTGS2/RELA/TLR4/TNF
hsa04510	Focal adhesion	1.056*E* − 11	15	CCND1/CTNNB1/EGFR/ERBB2/FYN/MET/PDGFRB/MAPK8/SRC/IL-6/STAT3/MAPK14/KIT/CDK4/PTPN11
hsa05144	Malaria	1.676*E* − 08	5	ICAM1/IL-6/MET/TLR4/TNF
hsa05416	Viral myocarditis	2.401*E* − 08	8	ABL1/CCND1/CASP3/FYN/ICAM1/LCK/PTPN11/TNF
hsa05220	Chronic myeloid leukemia	6.165*E* − 08	10	ABL1/CCND1/CDK4/PTPN11/RELA/CASP3/SRC/STAT3/KIT/PTGS2
hsa04915	Estrogen signaling pathway	6.51*E* − 08	6	EGFR/ESR1/HSP90AA1/MMP2/MMP9/SRC
hsa05202	Transcriptional misregulation in cancer	4.888*E* − 07	6	IL-6/MET/MMP9/PLAU/PPARG/RELA
hsa05130	Pathogenic *Escherichia coli* infection	1.25*E* − 06	7	ABL1/CTNNB1/FYN/TLR4/MET/PTPN11/SRC
hsa05210	Colorectal cancer	1.777*E* − 06	7	CCND1/CASP3/CTNNB1/MAPK8/PPARG/ESR1/SRC
hsa05014	Amyotrophic lateral sclerosis (ALS)	5.683*E* − 05	5	CASP3/MAPK14/TNF/APP/PTGS2
hsa04550	Signaling pathways regulating pluripotency of stem cells	0.0014409	3	MAPK14/CTNNB1/STAT3

**Table 4:** Visualization of the representative molecular docking results.

Target	Compound	Affinity (kcal/mol)	Zoomed complex	Complex
IL-6	Asiatic acid	−7.6	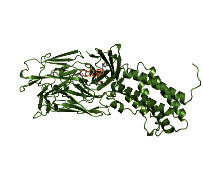	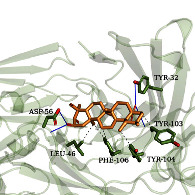
SRC	Asiatic acid	−8.7	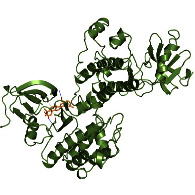	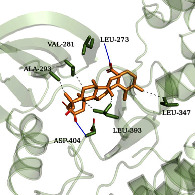
CTNNB1	Pratensein	−6.4	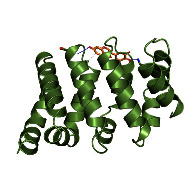	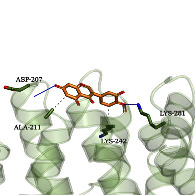
STAT3	Asiatic acid	−6.4	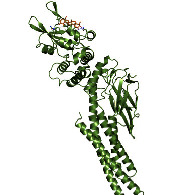	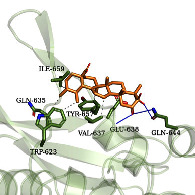
CASP3	Asiatic acid	−8.7	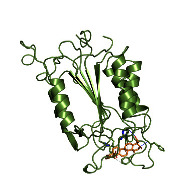	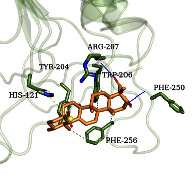
TNF	Sinapinic acid	−7.1	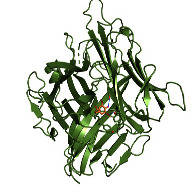	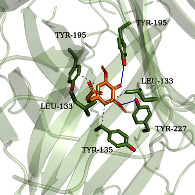
EGFR	Glycitein	−8.2	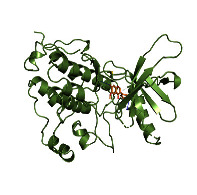	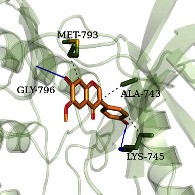
MAPK8	Pratensein	−9.0	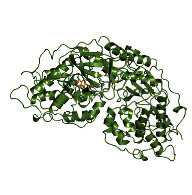	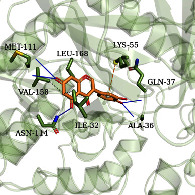
HSP90AA1	Ferulic acid	−7.1	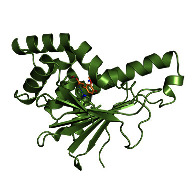	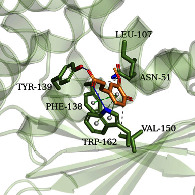
PTGS2	Glycitein	−7.1	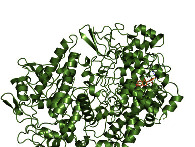	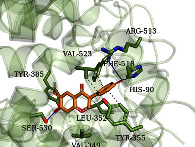

**Table 5:** Molecular docking of the known inhibitors with core targets.

Inhibitor	CAS ID	Target	Affinity (kcal/mol)
LMT-28	1239600-18-0	IL-6	−5.7
Src inhibitor 1	179248-59-0	SRC	−8.2
PNU 74654	113906-27-7	CTNNB1	−5.8
Static	19983-44-9	STAT3	−5.4
TNF-*α*-IN-1	444287-49-4	TNF	−9.4
EGFR inhibitor	879127-07-8	EGFR	−8.6
VER-50589	747413-08-7	HSP90AA1	−8.4
BUR1	23000-46-6	PTGS2	−6.2

## Data Availability

The data used to support the findings of this study are included within the article.

## Abbreviations

NSCLC: Non-small cell lung cancer
ZQFZ: Zhenqi Fuzheng
TCM: Traditional Chinese medicine
AsR: *Astragali Radix*
LLF: *Ligustri Lucidi Fructus*
UPLC: Ultra-performance liquid chromatography
RO5: Rule of five
Hdon: Hydrogen bond donors
Hacc: Hydrogen bond acceptors
LogP: Lipid-water partition coefficient
Rbon: Rotatable bonds
PPI: Protein–protein interaction
MCC: Maximal clique centrality
GO: Gene ontology
KEGG: Kyoto Encyclopedia of Genes and Genomes
BP: Biological process
CC: Cellular component
MF: Molecular function
LUAD: Lung adenocarcinoma
LUSD: Lung squamous cell carcinoma
OMIM: Online Mendelian Inheritance in Man
TTD: Therapeutic target database.

## Supplementary Materials

Supplementary Materials Table S1. The 106 common targets of ZQFZ granule against NSCLC. Table S2. Top 5 BP, CC, and MF pathway terms enriched in ZQFZ granule against NSCLC. Table S3. Molecular docking of core targets with top 5 compounds ranked by degree. Table S4. Structures of the known inhibitors for the core targets.
