# Supplementary material for: Multitarget and Multipathway Regulation of Zhenqi Fuzheng Granule against Non-Small Cell Lung Cancer Based On Network Pharmacology and Molecular Docking
Source: Evid Based Complement Alternat Med. 2022 Nov 17;2022:5967078. doi: 10.1155/2022/5967078 (PMC9691308; doi:10.1155/2022/5967078)
Supplement: Supplementary Materials — Table S1. The 106 common targets of ZQFZ granule against NSCLC. Table S2. Top 5 BP, CC, and MF pathway terms enriched in ZQFZ granule against NSCLC. Table S3. Molecular docking of core targets with top 5 compounds ranked by degree. Table S4. Structures of the known inhibitors for the core targets. [file 5967078.f1.docx]

Table S1. The 106 common targets of ZQFZ granule against NSCLC.

CA9

CA6

ADA

PNP

PTPN1

ACE

MME

ITGAL

ICAM1

ITGB2

CASP3

MMP13

MMP12

KDM4C

CAD

PARP1

PTPRC

TYMS

APEX1

COMT

DUSP1

CDC25C

CDC25A

EGFR

ALOX5

MMP9

MMP1

MMP2

APP

NFE2L2

STAT3

ESR2

TLR4

MET

CYP1A1

CYP1A2

CYP1B1

PTGS2

TTR

RELA

FYN

LCK

TLR9

AKR1B10

F3

NOS2

CCND1

CDK4

ABCB1

TOP2A

AHR

IGFBP3

ESR1

CTNNB1

F2

MAPK8

ILK

ECE1

CYP19A1

ERBB2

PIN1

TNF

IL2

ALDH2

TOP1

HSP90AA1

MIF

HSD17B1

ALOX12

PPARA

ABCG2

ABCC1

PON1

PLAT

PLAU

MCL1

IGFBP2

IGFBP1

SLC29A1

ABL1

KIT

PDGFRA

PDGFRB

MAPK14

SRC

MGMT

HSP90B1

ERCC5

RAF1

ACP1

CD81

PTGES

TERT

AR

PTPN6

PREP

PPARG

HMGCR

NR3C2

FABP4

PTPN11

NR3C1

CYP17A1

NR1H4

IL6

PTGER4

Table S2. Top 5 BP, CC and MF pathway terms enriched in ZQFZ granule against NSCLC.

| Type | Term | Pathway | pvalue | Count | Symbols |
| --- | --- | --- | --- | --- | --- |
| BP | GO:0006979 | response to oxidative stress | 8.951E-24 | 23 | ABL1/PARP1/APP/CASP3/CTNNB1/EGFR/FYN/IL6/MCL1/MET/MMP2/MMP9/PDGFRB/MAPK8/PTGS2/PTPN11/RELA/SRC/TLR4/CCND1/CDK4/ICAM1/KIT |
| BP | GO:0001934 | positive regulation of protein phosphorylation | 6.425E-23 | 31 | ABL1/APP/CCND1/ACE/EGFR/ERBB2/FYN/HSP90AA1/ICAM1/IL2/IL6/KIT/MMP9/PDGFRB/PPARG/PTSG2/PTPN11/PTPRC/SRC/TLR4/TNF/LCK/MET/MAPK14/PLAU/STAT3/AR/CTNNB1/MAPK8/CASP3/ESR1 |
| BP | GO:0007169 | transmembrane receptor protein tyrosine kinase signaling pathway | 5.065E-21 | 25 | ABL1/AR/CASP3/CDK4/MAPK14/CTNNB1/EGFR/ERBB2/FYN/KIT/LCK/MET/MMP2/MMP9/PDGFRB/PTPN11/RELA/SRC/STAT3/PARP1/APP/NR3C1/STAT3/MCL1/MET/PPARA/PPARG/TLR4 |
| BP | GO:0050727 | regulation of inflammatory response | 9.728E-20 | 23 | APP/MAPK14/EGFR/ESR1/FYN/IL2/IL6/MMP9/PPARA/PPARG/PTGS2/PTPRC/RELA/SRC/TLR4/TNF/HSP90AA1/PTPN11/ABL1/KIT |
| BP | GO:0010942 | positive regulation of cell death | 1.381E-19 | 18 | ABL1/PARP1/CASP3/CDK4/CTNNB1/FYN/IL6/LCK/MCL1/MMP9/PDGFRB/PPARG/MAPK8/PTGS2/PTPRC/SRC/TLR4/TNF |
| CC | GO:0045121 | membrane raft | 4.108E-14 | 14 | APP/CASP3/CTNNB1/EGFR/FYN/ICAM1/LCK/PTGS2/PTPN11/PTPRC/SRC/TNF/PDGFRB/PLAU |
| CC | GO:0043235 | receptor complex | 3.344E-09 | 10 | AHR/APP/EGFR/ERBB2/IL6/KIT/MET/PDGFRB/PPARG/TLR4 |
| CC | GO:0098552 | side of membrane | 1.756E-08 | 10 | ACE/FYN/ICAM1/KIT/LCK/ABCB1/PTPRC/SRC/TLR4/TNF |
| CC | GO:0048471 | perinuclear region of cytoplasm | 7.736E-08 | 15 | ABL1/APP/CDK4/CTNNB1/EGFR/ERBB2/FYN/HSP90AA1/SRC/TLR4/PDGFRB/ABCB1/MET/CASP3/IL6 |
| CC | GO:0005667 | transcription regulator complex | 1.252E-07 | 8 | PARP1/AHR/CCND1/CDK4/CTNNB1/PPARG/RELA/STAT3 |
| MF | GO:0019901 | protein kinase binding | 2.23E-17 | 17 | ABL1/PARP1/CCND1/MAPK14/CTNNB1/ACE/EGFR/ESR1/NR3C1/HSP90AA1/LCK/PDGFRB/PTPN11/PTPRC/RELA/SRC/STAT3 |
| MF | GO:0004714 | transmembrane receptor protein tyrosine kinase activity | 1.795E-13 | 16 | ABL1/EGFR/ERBB2/FYN/KIT/LCK/MET/PDGFRB/SRC/CCND1/CDK4/MAPK14/MAPK8/CTNNB1/PPARA/STAT3 |
| MF | GO:0019904 | protein domain specific binding | 6.076E-13 | 15 | ABL1/APP/AR/CTNNB1/FYN/HSP90AA1/KIT/LCK/MCL1/PPARA/PPARG/PTPN11/RELA/SRC/ERBB2 |
| MF | GO:0004879 | nuclear receptor activity | 1.03E-12 | 10 | AHR/AR/ESR1/NR3C1/PPARA/PPARG/STAT3/RELA/CTNNB1/EGFR |
| MF | GO:0061629 | RNA polymerase II-specific DNA-binding transcription factor binding | 1.314E-11 | 13 | PARP1/AR/MAPK14/CTNNB1/ESR1/PPARA/PPARG/RELA/SRC/STAT3/AHR/CCND1/EGFR |

Table S3. Molecular docking of core targets with top 5 compounds ranked by degree.

| **Compound** | **Target** | **PDB ID** | **Resolution (Å)** | **Affinity (kcal/mol)** |
| --- | --- | --- | --- | --- |
| Sinapinic acid | IL6 | 4cni | 2.20 | -4.7 |
| Sinapinic acid | SRC | 4mxo | 2.10 | -5.8 |
| Sinapinic acid | CTNNB1 | 7afw | 1.814 | -4.5 |
| Sinapinic acid | STAT3 | 6njs | 2.70 | -4.8 |
| Sinapinic acid | CASP3 | 3kjf | 2.00 | -5.3 |
| Sinapinic acid | TNF | 7jra | 2.10 | -7.1 |
| Sinapinic acid | EGFR | 4i23 | 2.80 | -5.8 |
| Sinapinic acid | MAPK8 | 2no3 | 3.20 | -3.4 |
| Sinapinic acid | HSP90AA1 | 5cf0 | 1.80 | -6.1 |
| Sinapinic acid | PTGS2 | 5f1a | 2.38 | -6.3 |
| Ferulic acid | IL6 | 4cni | 2.20 | -5.1 |
| Ferulic acid | SRC | 4mxo | 2.10 | -5.9 |
| Ferulic acid | CTNNB1 | 7afw | 1.814 | -4.6 |
| Ferulic acid | STAT3 | 6njs | 2.70 | -4.9 |
| Ferulic acid | CASP3 | 3kjf | 2.00 | -5.6 |
| Ferulic acid | TNF | 7jra | 2.10 | -7.2 |
| Ferulic acid | EGFR | 4i23 | 2.80 | -6.2 |
| Ferulic acid | MAPK8 | 2no3 | 3.20 | -6.3 |
| Ferulic acid | HSP90AA1 | 5cf0 | 1.80 | -7.1 |
| Ferulic acid | PTGS2 | 5f1a | 2.38 | -6.9 |
| Asiatic acid | IL6 | 4cni | 2.20 | -7.6 |
| Asiatic acid | SRC | 4mxo | 2.10 | -8.7 |
| Asiatic acid | CTNNB1 | 7afw | 1.814 | -5.9 |
| Asiatic acid | STAT3 | 6njs | 2.70 | -6.4 |
| Asiatic acid | CASP3 | 3kjf | 2.00 | -8.7 |
| Asiatic acid | TNF | 7jra | 2.10 | 0.0 |
| Asiatic acid | EGFR | 4i23 | 2.80 | -7.5 |
| Asiatic acid | MAPK8 | 2no3 | 3.20 | -4.5 |
| Asiatic acid | HSP90AA1 | 5cf0 | 1.80 | -7.3 |
| Asiatic acid | PTGS2 | 5f1a | 2.38 | 15.6 |
| Pratensein | IL6 | 4cni | 2.20 | -6.2 |
| Pratensein | SRC | 4mxo | 2.10 | -8.1 |
| Pratensein | CTNNB1 | 7afw | 1.814 | -6.4 |
| Pratensein | STAT3 | 6njs | 2.70 | -6.3 |
| Pratensein | CASP3 | 3kjf | 2.00 | -7.8 |
| Pratensein | TNF | 7jra | 2.10 | -8.9 |
| Pratensein | EGFR | 4i23 | 2.80 | -8.2 |
| Pratensein | MAPK8 | 2no3 | 3.20 | -9.0 |
| Pratensein | HSP90AA1 | 5cf0 | 1.80 | -8.3 |
| Pratensein | PTGS2 | 5f1a | 2.38 | -6.6 |
| Glycitein | IL6 | 4cni | 2.20 | -6.0 |
| Glycitein | SRC | 4mxo | 2.10 | -8.1 |
| Glycitein | CTNNB1 | 7afw | 1.814 | -5.9 |
| Glycitein | STAT3 | 6njs | 2.70 | -6.1 |
| Glycitein | CASP3 | 3kjf | 2.00 | -7.4 |
| Glycitein | TNF | 7jra | 2.10 | -8.9 |
| Glycitein | EGFR | 4i23 | 2.80 | -8.2 |
| Glycitein | MAPK8 | 2no3 | 3.20 | -8.3 |
| Glycitein | HSP90AA1 | 5cf0 | 1.80 | -8.0 |
| Glycitein | PTGS2 | 5f1a | 2.38 | -7.1 |

Table S4. Structures of the known inhibitors for the core targets.

| **Inhibitor** | **CAS ID** | **Target** | **Structural formula** |
| --- | --- | --- | --- |
| LMT-28 | 1239600-18-0 | IL6 | 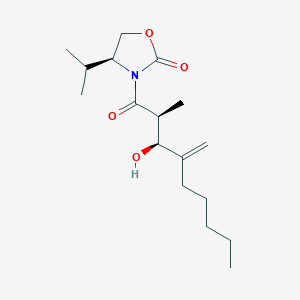 |
| Src Inhibitor 1 | 179248-59-0 | SRC | 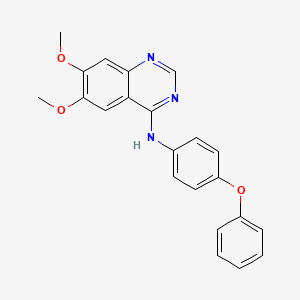 |
| PNU 74654 | 113906-27-7 | CTNNB1 | 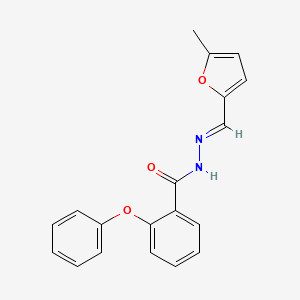 |
| Stattic | 19983-44-9 | STAT3 | 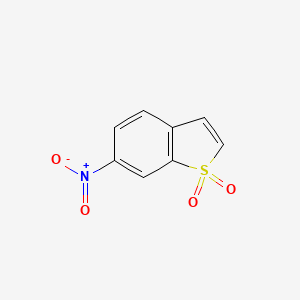 |
| TNF-α-IN-1 | 444287-49-4 | TNF | 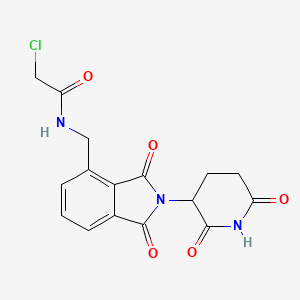 |
| EGFR inhibitor | 879127-07-8 | EGFR | 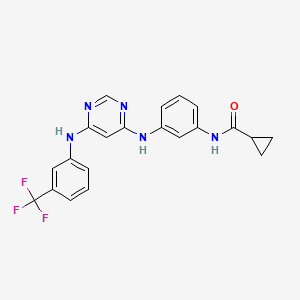 |
| VER-50589 | 747413-08-7 | HSP90AA1 | 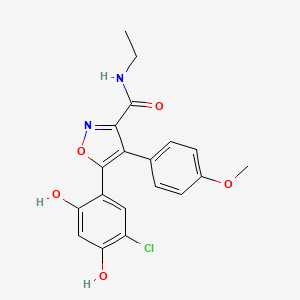 |
| BUR1 | 23000-46-6 | PTGS2 | 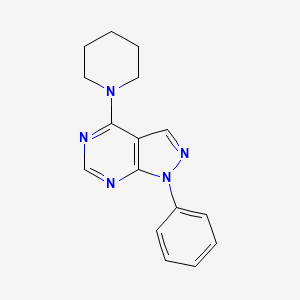 |
